# Role of hypoxia-inducible factor 1alpha in the integrity of articular cartilage in murine knee joints

**DOI:** 10.1186/ar2508

**Published:** 2008-09-12

**Authors:** Kolja Gelse, David Pfander, Simon Obier, Karl X Knaup, Michael Wiesener, Friedrich F Hennig, Bernd Swoboda

**Affiliations:** 1 Division of Orthopaedic Rheumatology, University of Erlangen-Nuremberg, Rathsberger Straße 57, Erlangen 91054, Germany; 2 Department of Orthopaedic Trauma Surgery, University Hospital Erlangen, Krankenhausstraße 12, Erlangen 91054, Germany; 3 Interdisciplinary Center for Clinical Research, University Hospital Erlangen, Glückstraße 6, Erlangen 91054, Germany; 4 Division of Orthopaedic Rheumatology, Hufeland-Clinic, Langensalzaer Landstraße 1, Muehlhausen 99974, Germany

## Abstract

**Introduction:**

Chondrocytes have to withstand considerable hypoxic conditions within the avascular articular cartilage. The present study investigated the effects of inhibiting or stabilizing hypoxia-inducible factor (HIF)-1α by 2-methoxyestradiol or dimethyloxaloylglycine on the progression of osteoarthritis in murine knee joints.

**Methods:**

2-Methoxyestradiol was injected six times over a period of 2 weeks into the left knee joint of Balb/C mice. Joints were assessed by histochemical and immunohistochemical methods, 3 weeks and 12 weeks following the first injection. Dimethyloxaloylglycine, an inhibitor of HIF-degrading prolyl-hydroxylases, was injected into the left knee joints of STR/ORT mice once a week over the entire period of 12 weeks. Right knee joints that received a saline solution served as controls. In addition, the effects of dimethyloxaloylglycine on HIF-1 target gene expression and on collagen metabolism were analyzed *in vitro*.

**Results:**

Injection of 2-methoxyestradiol led to osteoarthritic changes in the treated knee joints of Balb/C mice. The first signs of osteophyte formation were observed in the knee joints after 3 weeks, followed by progressive destruction of the articular cartilage at 12 weeks that was not, however, accompanied by inflammatory reactions. Injection of dimethyloxaloylglycine could not prevent severe osteoarthritis that spontaneously developed in the knee joints of STR/ORT mice. In chondrocyte cultures, administration of dimethyloxaloylglycine resulted in an upregulation of Sox9 expression. Such a stimulatory effect was not observed, however, for the expression of type II collagen, which might be the indirect consequence of intracellular collagen retention observed by immunofluorescence or of increased expression of IL-1β and IL-6.

**Conclusions:**

Induction of osteoarthritis by 2-methoxyestradiol demonstrates the importance of HIF-1 in maintaining the integrity of hypoxic articular cartilage. Stabilization of HIF-1 by dimethyloxaloylglycine, however, was not of therapeutic value, since this nonselective prolyl-hydroxylase inhibitor also interferes with proper collagen metabolism and induces the expression of catabolic cytokines

## Introduction

Articular cartilage is a unique connective tissue that physiologically lacks blood vessels. This lack of vessels inevitably coincides with a significantly reduced oxygen level within the tissue, which requires well-adapted mechanisms to ensure survival of the resident cells. The transcription factor hypoxia-inducible factor (HIF)-1 represents one important element in maintaining proper cellular functions under such hypoxic conditions [1]. As for chondrocytes, HIF-1 is also of great importance by promoting the synthesis of relevant extracellular matrix components [2]. This synthesis may, at least partly, be mediated by transactivation of Sox9, a key transcription factor for many cartilage-specific genes involving metabolism and chondrogenic differentiation [3,4].

The importance of HIF-1 for the formation and maintenance of cartilage tissue has been demonstrated in conditional knockout mice in which deletion of its oxygen-sensitive subunit HIF-1α severely interfered with proper skeletal development and led to massive cell death within the center of the forming cartilaginous elements [1]. On the contrary, another study in mice with conditional inactivation of the von Hippel–Lindau protein demonstrated that the resulting stabilization of HIF-1α by inhibiting its degradation increased the deposition of extracellular cartilage matrix in the growth plate [5].

The regulation of HIF-1 activity is complex. Under normoxic conditions, HIF-1α is degraded rapidly. In the presence of molecular oxygen, two prolyl residues within the oxygen-dependent degradation domain of the HIF-1α protein are hydroxylated by HIF-specific oxygen-dependent prolyl-hydroxylases [6]. This conversion allows capture by the von Hippel–Lindau protein complex followed by ubiquitinylation and rapid degradation by the proteasome [6,7]. The synthesis of HIF-1α can be activated by a variety of factors, including reactive oxygen species, glucose metabolites, and a number of growth factors or cytokines involving the phosphatidylinositol 3-kinase or extracellular signal-regulated kinase/mitogen-activated protein kinase pathway [8-10].

In osteoarthritic cartilage, the protein levels of HIF-1 are significantly increased and its activity correlates to the severity of degenerative cartilage changes [9,11]. According to the biological functions of HIF-1, it may be assumed that HIF-1 exerts a compensatory protective role in the disease process rather than promoting the progression of the disease.

To further prove this hypothesis, we established two animal models. The first model served to investigate whether inhibition of HIF by 2-methoxyestradiol (2ME2) promotes or initiates osteoarthritis (OA) in the murine knee joint. In contrast to conditional knockout mice, the chemical inhibition allows one to investigate the effects in adult joints in an otherwise healthy organism, and therefore seems better suited referring to studies on OA. Although the exact mechanism of HIF inhibition by 2ME2 has still to be defined, 2ME2 has been shown to reliably decrease the levels of HIF-1α protein in chondrocytes and a number of other cell types – and as a consequence also decreases the expression of a number of HIF-1 target genes including phosphoglycerate kinase 1 (PGK1), vascular endothelial growth factor A, and glucose transporter type 1 [12-14].

The second animal model was used to investigate whether stabilization of HIF-1α by dimethyloxaloylglycine (DMOG) prevents or delays degenerative changes in STR/ORT mice. DMOG is known to efficiently inhibit prolyl-hydroxylases that mediate the oxygen-dependent degradation of HIF [15]. STR/ORT mice served as an OA model since this strain spontaneously develops degenerative changes in the knee joints [16-19].

## Materials and methods

### Cell isolation and culture

Primary human chondrocytes were isolated from knee cartilage of six patients undergoing total knee replacement for OA as described previously [12]. All of the samples were obtained after the patients gave their informed consent. Our institutional ethics committee approved the study protocol.

To minimize the effect of chondrocyte dedifferentiation, only first-passage chondrocytes were used in the present study. After digestion, cells were plated at a cell density of 100,000 cells/cm^2 ^in 24-well plates for RNA isolation or in Flexi-Perm culture chambers (Greiner Bio-One, Solingen, Germany) for immunohistochemistry.

First, the cells were cultured under normoxic conditions (21% oxygen) for 3 days in DMEM containing 10% FCS, 2 mM glutamine and 50 U/ml streptomycin/penicillin at 37°C. For gene expression analyses, confluent cells were subsequently treated with different concentrations of DMOG ranging from 0.1 to 1 mM or with 2ME2 in concentrations of 100 or 200 μM, and were cultured either under normoxic (21% oxygen) or hypoxic conditions (1% oxygen) for 24 hours before harvesting. Since 2ME2 is dissolved in dimethyl sulfoxide (DMSO), a portion of the cells was exposed to 1% DMSO alone, which served as an additional control. For hypoxic conditions, the chondrocytes were kept in a sealed incubator (Binder GmbH, Tuttlingen, Germany), and flushed with gas mixture containing 1% oxygen, 5% carbon dioxide and balanced with 94% N_2 _in a humidified atmosphere. For immunohistochemistry, the cells were treated with 1 mM DMOG and were cultured under normoxic conditions for 5 days.

### Animal experiments

The present study investigated the effect of 2ME2 on female Balb/C mice (Charles River, Sulzfeld, Germany) and the effect of DMOG on female STR/ORT mice (Harlan Winkelmann, Borchen, Germany). The animals were fed a standard laboratory diet *ad libitum *and were allowed to move freely in their cages at any time. For all procedures, the mice were anesthetized by inhalation with 1.5 l/min isofluran (Baxter, Unterschleißheim, Germany) and 1.5 l/min oxygen.

In 14-week-old Balb/C-mice (n = 32), 2ME2 (20 μl) at concentrations of 10 μM (group 1, n = 16) or 100 μM (group 2, n = 16) was carefully injected with a 27G needle into the knee joints through the patella tendon into the intercondylar notch region to avoid contact with the articular surface. The treatment was repeated every other day for a total of six injections during the course of the first 2 weeks of the experiment. The animals received no further treatment for the remaining 1 week or 10 weeks, respectively. A control solution (20 μl) was injected into the right knee joints of all Balb/C mice at the same time. Since 2ME2 was dissolved in a 1% DMSO saline solution, the control solution (0.9% NaCl) was also supplemented with 1% DMSO. Preliminary experiments *in vitro *excluded any toxic effects of 1% DMSO. The mice were killed either after 3 weeks (n = 8 per group) or 12 weeks (n = 8 per group) by cervical dislocation.

In 8-week-old STR/ORT mice (n = 8), 20 μl of a 1 mM DMOG solution were injected into the left knee joints once per week throughout the whole period of 12 weeks. DMOG was dissolved in 0.9% NaCl. As a control, 20 μl saline solution was injected into the right knee joints at the same time. The STR/ORT mice were sacrificed after 12 weeks.

All knee joints were fixed in 4% paraformaldehyde for 12 hours and were decalcified in 0.5 M ethylenediamine tetraacetic acid/2% paraformaldehyde for 2 weeks. After standard processing, the samples were embedded in paraffin. Serial frontal 5 μm sections of the knee joints were cut and further processed for histological and immunohistological analysis.

All procedures on the animals were approved by the appropriate institutional Review board.

### Histological assessment

After deparaffinization, serial sections were stained with toluidine blue or with H&E for further histological investigation. Sections from treated joints and control joints were compared by scoring systems investigating the articular cartilage layer, osteophyte formation and synovial tissue.

The articular cartilage layer was evaluated referring to a mouse-specific scoring system described by Walton [16] (grade 0 = normal; grade 1 = superficial fibrillation, alterations in proteoglycan staining; grade 2 = deeper fissures, beginning loss of cartilage tissue; grade 3 = substantial loss of uncalcified cartilage tissue, fissures extending to the subchondral bone; grade 4 = complete loss of cartilage tissue, exposure of bone).

Staging of osteophyte formation was analyzed according to a previously described system [20] (grade 0 = normal; grade 1 = fibrous outgrowths; grade 2 = fibrocartilaginous tissue; grade 3 = mature cartilage tissue; grade 4 = cellular hypertrophy, bone core).

The evaluation of the synovial layer refers to the additive score by Krenn and colleagues [21], including thickening of the synovial cell lining (grade 0 = single cell layer; grade 1 = two to three cell layers; grade 2 = four to five cell layers; grade 3 = more than five cell layers), synovial inflammation (grade 0 = no inflammation; grade 1 = single lymphocytes; grade 2 = lymphocytic aggregations; grade 3 = formation of lymph follicles) and the cell density of the synovial stroma (grade 0 = normal cell density; grade 1 = slightly increased cellular density; grade 2 = increased cellular density, 3 = high cellular density, presence of multinuclear giant cells).

The sections were analyzed blind, by three independent experts.

### Detection of cellular hypoxia

Pimonidazole hydrochloride 60 mg/kg (Hydroxyprobe™-1 Plus Kit; Chemicon, Temecula, CA, USA) was injected intraperitoneally in Balb/C mice (n = 6). The animals were sacrificed after 24 hours. The knee joints and other organs were fixed in 4% paraformaldehyde for 12 hours and were processed as described above. Representative sections were immunostained using a FITC-conjugated monoclonal antibody (Hydroxyprobe™-1 Mab1; Chemicon). Immunostaining was performed according to the manufacturer's recommendations. Antibody binding was detected by a peroxidase-labeled secondary antibody against FITC and was visualized by diaminobenzidine tetrahydrochloride (Chemicon, Temecula, CA, USA). All sections were counterstained with hematoxylin.

### Immunohistochemistry

For detection of type II collagen, sections were pretreated with 0.2% hyaluronidase (Roche, Mannheim, Germany) in PBS (pH 5.0) for 60 minutes, and subsequently treated with pronase (2 mg/ml in PBS, pH 7.3; Sigma-Aldrich, Munich, Germany) for 60 minutes at 37°C. No enzymatic pretreatment of the sections was required for detection of CD45. Nonspecific antibody binding was blocked with 5% BSA in PBS.

For signal amplification and visualization of HIF-1α, an amplification system (CSA kit; Dako, Hamburg, Germany) was used according to the manufacturer's instructions. Antigen retrieval was performed for 6 minutes in preheated Dako target retrieval solution, using a pressure cooker.

The slides were incubated overnight at 4°C with polyclonal rabbit antibodies against HIF-1α (Cayman Chemical, Ann Arbor, MI, USA) in a 1:10,000 dilution, with monoclonal mouse anti-human type II collagen antibodies (MP Biomedicals, Irvine, CA, USA) in a 1:500 dilution or with monoclonal rat anti-mouse CD45 antibodies (BD Pharmingen, Heidelberg, Germany) in a 1:200 dilution, followed by washing with Tris-buffered saline. For detection of type II collagen or CD45, the sections were subsequently incubated with a biotinylated donkey anti-mouse secondary antibody (Dianova, Hamburg, Germany) or a biotinylated rabbit anti-rat secondary antibody (Vector Laboratories, Burlingame, CA, USA), followed by treatment with a complex of streptavidin and biotinylated alkaline phosphatase. The sections were developed with fast red and were counterstained with hematoxylin.

For visualization of HIF-1α, a catalyzed signal amplification kit (Dako) based on a streptavidin–biotin–peroxidase reaction was used. 3,3'-Diaminobenzidine served as the chromogen for the peroxidase reaction.

### Immunofluorescence

After fixation of the cultured cells with 70% ethanol, the slides were blocked with 5% BSA and were incubated with monoclonal mouse anti-human type II collagen antibodies (MP Biomedicals) in a 1:500 dilution for 50 minutes at 37°C. After washing with PBS, the cells were incubated with a Cy3-conjugated anti-mouse antibody (Dianova) diluted at a ratio of 1:100 for 45 minutes. After washing with Tris-buffered saline, the slides were covered with a mounting medium containing 4',6'-diamidino-2-phenylindole (Vector, Peterborough, UK) and were analyzed by fluorescence microscopy. Control slides were incubated with equivalent concentrations of mouse IgG.

### TUNEL staining

For the detection of *in situ *DNA breaks, the TUNEL reaction was applied using the In Situ Cell Death Detection Kit, AP (Roche, Mannheim, Germany). The proteinase K pretreatment as well as the terminal deoxynucleotidyl transferase concentrations were carefully titrated to allow sensitive and specific detection of apoptotic cell nuclei. After deparaffinization, the sections were washed in PBS and digested with proteinase K (20 μg/ml; Boehringer, Ingelheim, Germany) for 15 minutes at 37°C. After washing with PBS, sections were incubated with terminal deoxynucleotidyl transferase solution in reaction buffer at a volume ratio of 9:1 at 37°C for 1 hour followed by extensive washing. The converter AP antibody was added for 30 minutes at 37°C. After washing, detection was performed by fast red staining and counterstaining with hematoxylin.

### RNA isolation and real-time RT-PCR

The expression levels of Sox9, type II collagen α_1_-chain (Col2A1), type I collagen α_2_-chain (Col1A2), PGK1, IL-1β, IL-6 and TNFα under the influence of different concentrations of DMOG or 2ME2 were compared for normoxic or hypoxic conditions by real-time RT-PCR using RNA preparations from cultured primary human chondrocytes of three different donors. Total RNA was isolated from the cells by the Nucleo-Spin-RNA-II-Kit (Clontech Laboratories, Mountain View, CA, USA). Quantitative real-time RT-PCR was performed with an ABI Prism 7900 sequence detection system (Applied Biosystems, Foster City, CA, USA) and a QuantiTect Probe RT-PCR Kit (Qiagen, Chatsworth, CA, USA) for one-step RT-PCR.

The relative quantification of gene expression was performed using the standard curve method. For each sample, the relative amount of the target mRNA was determined and normalized to β2-microglobulin mRNA.

The primer and probe sets for Sox9, Col1A2, Col2A1, IL-1β, IL-6 and TNFα were purchased from Applied Biosystems and from Celera Genomics (Rockville, MD, USA). The primer and probe sets for β_2_-microglobulin and PGK1 were designed by the primer express software (PerkinElmer, Emeryville, CA, USA) as described previously [13].

### Statistical analysis

All data are presented as the mean ± standard deviation. The analysis of the morphological scores was performed using the Mann–Whitney test. The results of quantitative gene expression were analyzed using a two-sided Student's *t *test. *P *<0.05 was considered significant.

## Results

### Tissue hypoxia in articular cartilage and meniscal tissue

**Figure 1:**
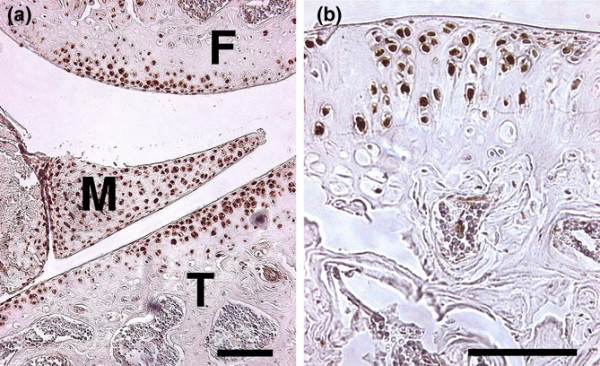
Detection of tissue hypoxia. Pimonidazole hydrochloride (Hydroxyprobe™-1 solution; Chemicon) was injected intraperitoneally in Balb/C mice and detected in hypoxic cells immunohistochemically after 24 hours. Most **(a), (b) **articular chondrocytes and (a) meniscal cells stained positive for this hypoxic marker, whereas osteocytes and other mesenchymal cells were negative. F, femoral condyle; M, meniscus; T, tibial plateau. Bars = 100 μm.

The intraperitoneal injection of pimonidazole hydrochloride in Balb/C mice allowed investigation of intracellular hypoxia. Pimonidazole is a 2-nitroimidazole that is reductively activated and becomes covalently bound to thiol-containing proteins only in hypoxic cells [22]. Immunohistochemical detection of pimonidazole–protein adducts revealed strong staining in articular chondrocytes and meniscal cells even in superficial layers (Figure 1a,b). Osteocytes, synoviocytes, muscle cells and cells of other tissues and organs were negative for this hypoxia marker.

### Repeated intraarticular injection of 2-methoxyestradiol induces osteoarthritic changes in Balb/C mice

**Figure 2:**
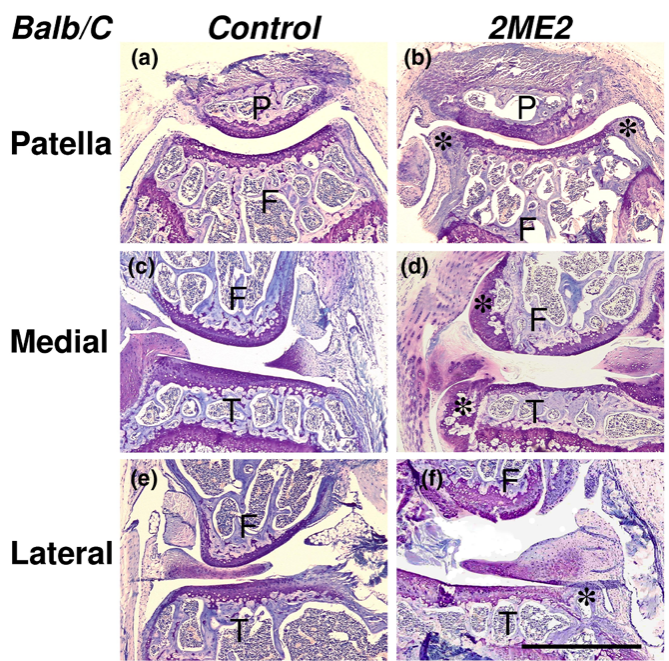
Histomorphology of treated knee joints of Balb/C mice. The joints were analyzed 12 weeks after the first intra-articular injection of **(a), (c), (e) **the control solution or **(b)**, **(d)**, (**f) **100 μM 2-methoxyestradiol (2ME2). In the control joints, representative coronar sections of (a) the patellofemoral joint, as well as (c) the medial knee compartment and (e) the lateral knee compartment, did not show relevant signs of cartilage degeneration or osteophyte formation. (c) The menisci remained intact. 2ME2-treated joints were characterized by significant osteophyte formation (asterisks) and degeneration of the articular cartilage in (b) the patellofemoral joint, as well as in the (f) lateral and (d) medial knee compartments. Toluidine blue staining. Bar = 1 mm. P, patella; F, femur; T, tibia.

**Figure 3:**
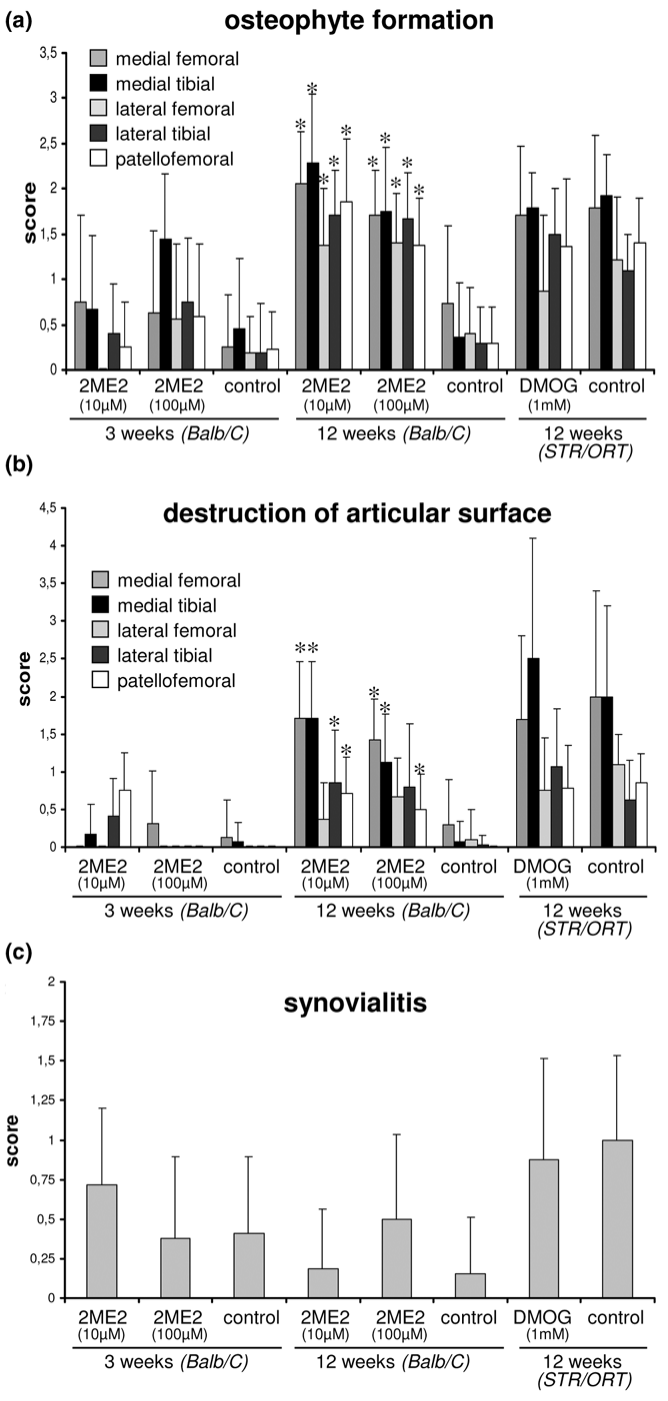
Osteophyte formation, destruction of the articular surface and synovial tissue analysis. Analysis of **(a) **osteophyte formation, **(b) **destruction of the articular surface and **(c) **analysis of synovial tissue. The knee joints were treated by administration of 2-methoxyestradiol (2ME2) in Balb/C mice or of dimethyloxaloylglycine (DMOG) in STR/ORT mice. Bars show the mean ± standard deviation. *P <0.05, **P <0.01 versus untreated control joint.

Two different concentrations of 2ME2 (group 1, 10 μM; group 2, 100 μM) were injected six times into the left knee joints over the period of the first 2 weeks. Contralateral right knee joints received a control solution. Coronar section of the patellofemoral joint as well as the medial and lateral compartment were assessed for degenerative changes of the articular cartilage, for osteophyte formation and for synovitis. In the control joints, the administration of the control solution (1% DMSO in 0.9% NaCl) did not induce degenerative changes of the articular cartilage or relevant osteophyte formation within the observation period of 12 weeks (Figures 2a,c,e and 3a,b).

The joints treated with 2ME2 were characterized at 3 weeks by beginning osteophyte formation and the first signs of degenerative changes of the articular cartilage layer. Osteophytes were still premature and were characterized by fibrous and fibrocartilaginous tissue lacking a vascularized bone core (data not shown). After 12 weeks, 2ME2-treated joints differed significantly from control joints with respect to degeneration of the articular cartilage and osteophyte formation (Figures 2b,d,f and 3a,b). The formation of osteophytes was present at the margins of the articular surface in all joint compartments. The articular cartilage showed signs of degeneration, with fissuring and loss of superficial layers of the matrix (Figure 2b,d,f). The menisci also revealed signs of degenerative changes with fraying and loss of tissue (Figure 2d). Luxation of the patella or other defective positions of joint structures was not observed.

**Figure 4:**
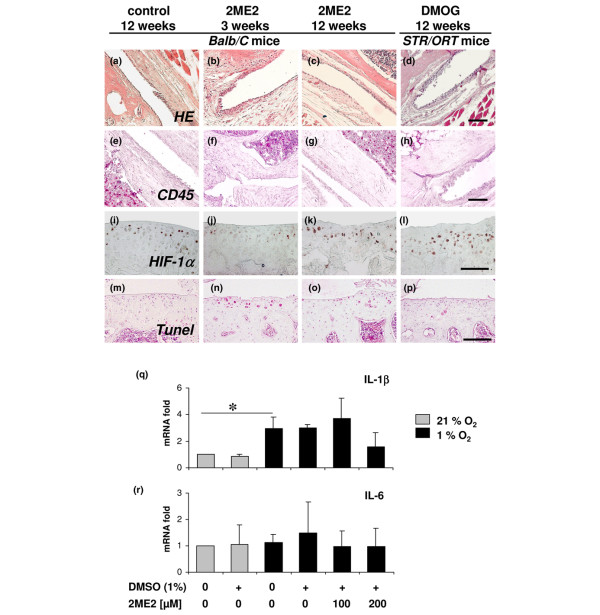
Effects of 2-methoxyestradiol and dimethyloxaloylglycine. Effects on synovial tissue, levels of hypoxia-inducible factor (HIF) 1α and viability of chondrocytes, and the effect of 2-methoxyestradiol (2ME2) on the expression of inflammatory cytokines. **(a) **to **(d) **Administration of 2ME2 (10 μM) or of dimethyloxaloylglycine (DMOG) (1 mM) did not induce inflammation or fibrosis of synovial tissue. A slight thickening of the synovial cell lining could be observed (b) 1 week following the repeated 2ME2 injections and (d) in osteoarthritic joints of STR/ORT mice. Immunostaining for CD45 revealed no relevant invasion of leukocytes into synovial tissue in **(e) **control joints, **(f)**, **(g) **2ME2-treated joints or **(h) **DMOG-treated joints. Large amounts of CD45-positive cells could be detected within bone marrow spaces. **(i) **Immunohistochemical detection of HIF-1α revealed strong staining in most articular chondrocytes of control joints of Balb/C mice. **(j) **2ME2-treated joints showed a distinct reduction of HIF-1α-positive cells with less intense staining at 3 weeks. **(k) **Number of positive cells recovered in joints analyzed at 12 weeks. **(l) **DMOG treatment did not relevantly influence the number of positive cells. **(m) **Apoptosis in articular cartilage, visualized by the TUNEL method, was virtually absent in the control joints. **(n) **Treatment with 2ME2 resulted in a considerable number of TUNEL-positive cells, particularly in superficial layers at 3 weeks. **(o) **After 12 weeks, the number of positive cells declined but was still elevated compared with controls. **(p) **Only few cells were TUNEL-positive in the articular cartilage of DMOG-treated STR/ORT mice. Expression of **(q) **IL-1β and **(r) **IL-6 in cultured articular chondrocytes determined by quantitative RT-PCR under 21% or 1% oxygen and treatment with or without 2ME2. Treatment with the solvent dimethyl sulfoxide alone served as an additional control. Expression levels are shown as the mean ± standard deviation. *P <0.05. Bars = 100 μm.

The analysis of the synovial membrane revealed slight thickening of the synovial lining up to three or four cell layers in both treated joints and control joints at 3 weeks (Figure 4b). After 12 weeks, however, this tendency declined and the synovial membrane was again lined with a single cell layer in most cases (Figure 4a,c). Signs of inflammation, increased cell density within the synovial stroma or fibrosis of the joint capsule could not be observed in any animal during the whole observation period (Figure 4a,b,c,e,f,g). The additive synovial score values of 2ME2-treated joints or control joints did not reach the level of low-grade synovitis (additive score value 1 to 3) (Figure 3c).

### Dimethyloxaloylglycine does not prevent the induction of osteoarthritis in STR/ORT mice

To investigate the hypothesis that therapeutic stabilization of HIF-1α in articular chondrocytes would prevent the progression of osteoarthritis in STR/ORT mice, we injected DMOG into the left knee joints once a week during the entire period of 12 weeks. Right knee joints received a 0.9% NaCl control solution at the same time. The joints were analyzed 12 weeks following the first injection.

**Figure 5:**
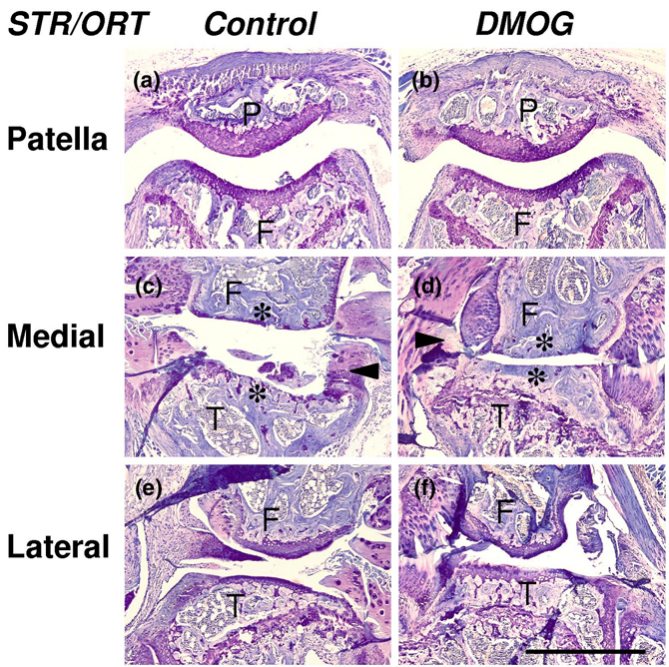
Histomorphology of treated knee joints of STR/ORT mice. The joints were analyzed 12 weeks after the first intra-articular injection of **(a)**, **(c)**, **(e) **the control solution or **(b)**, **(d)**, **(f) **1 mM dimethyloxaloylglycine (DMOG). In both control joints and treated joints, representative coronar sections of the (a), (b) patellofemoral joint, (c), (d) the medial knee compartment and (e), (f) the lateral knee compartment showed distinct signs of cartilage degeneration. There were no significant differences between both groups. (c), (d) Particularly, the medial femoral condyle and medial tibial plateau were affected with complete loss of the articular cartilage (asterisks) and degeneration of the meniscus (arrowheads) in both groups. P, patella; F, femur; T, tibia. Toluidine blue staining. Bar = 1 mm.

Twelve weeks after the first injection, the control joints of these 20-week-old mice showed distinct degenerative changes – as described before for this and related mouse strains [16,17,19]. The most prominent degenerative changes were observed in the medial compartment, with massive loss of the cartilage substance and meniscal tissue to the point of complete loss of cartilage tissue with exposure of the subchondral bone (Figure 5a,c,e).

The intraarticular administration of DMOG did not prevent such degenerative changes. We could not determine any significant positive or negative effect of DMOG with respect to degenerative changes of the articular cartilage (Figures 3a,b and 5b,d,f). Comparable with the control joints, the medial knee compartment showed massive degenerative changes culminating in a complete loss of articular cartilage tissue and degeneration of the menisci. The cartilage of the lateral compartment and the patellofemoral joint was affected with fibrillation and fissuring. There were also no significant differences between DMOG-treated joints and control joints with respect to the formation of osteophytes. Osteophytes were present in most cases at the margins of the joint surface of all compartments (Figure 5b,d,f). Luxation of the patella was not observed.

The analysis of the synovial membrane using the scoring system of Krenn and colleagues [21] did not reveal any significant differences between joints that received DMOG (0.87 ± 0.64) or the control solution (1.0 ± 0.54) (Figure 3c). The slightly elevated values could be ascribed to a moderate thickening of the synovial cell lining, which was not, however, invaded by lymphocytes or myeloid cells. An increased cellular density of the stroma was not observed (Figure 4d,h). The additive score values approximate the lower limit of low-grade synovitis (additive score value of 1 to 3), which is an often-observed phenomenon in advanced joint degeneration.

### Detection of HIF-1α levels and cellular apoptosis in articular cartilage

Preceding *in vitro *studies of our group have recently demonstrated the effect of 2ME2 on decreasing the protein level and functional activity of HIF-1α as well as the HIF-stabilizing effects of DMOG [12,13]. To prove the validity of the described effects *in vivo*, we investigated staining for HIF-1α immunohistochemically in treated joints and control joints. In control joints of both mouse strains, HIF-1α could clearly be detected in most articular chondrocytes throughout all layers (Balb/C, Figure 4i; STR/ORT, data not shown). The distribution pattern of HIF-1α-staining correlated with the staining for hypoxic cells with the Hydroxyprobe™ method (Chemicon). There was a distinct reduction of HIF-1α-positive cells at 3 weeks in joints that received 2ME2 (Figure 4j). At 12 weeks, the number of HIF-positive cells recovered and was comparable with control joints (Figure 4k). Following DMOG treatment, HIF-1α could strongly be detected in a high percentage of articular chondrocytes (Figure 4l). HIF-1α could also be detected in meniscal cells, but not in osteocytes, muscle cells, synoviocytes or fibroblasts of ligaments (data not shown).

To investigate whether the inhibition of HIF-1α in hypoxic cells induces apoptosis, we visualized *in situ *DNA breaks by the TUNEL method. Apoptosis was virtually absent in the control joints (Figure 4m). Following treatment with 2ME2, a considerable number of TUNEL-positive cells could be observed particularly in the superficial half of the articular cartilage layer at 3 weeks (Figure 4n), but not in other tissues such as bone, synovial membrane or muscles. After 12 weeks, the percentage of TUNEL-positive cells in the cartilage declined but was still elevated compared with control joints (Figure 4o). In STR/ORT mice, we could not detect any significant amounts of TUNEL-positive cells independent of the treatment with DMOG (Figure 4p) or with control solution (data not shown).

### 2-Methoxyestradiol does not induce the expression of inflammatory cytokines in articular chondrocytes

The effect of 2ME2 on the expression of IL-1β, IL-6 and TNFα in human articular chondrocytes was measured by quantitative RT-PCR. Cultivation under hypoxia (1% oxygen) significantly induced the expression of IL-1β compared with cultivation under normoxic conditions. Under hypoxic conditions, the inhibition of HIF-1α by administration of 100 μM 2ME2 did not alter IL-1β expression compared with hypoxic controls. The highest dose of 2ME2 (200 μM) led to a measurable, although not significant, decrease in IL-1β expression. As a control, the application of the solvent (1% DMSO) alone had no influence on the expression (Figure 4q).

The expression of IL-6 was not influenced by the administration of 2ME2 or DMSO irrespective of the oxygen status (Figure 4r). We could not detect any relevant expression of TNFα in the articular chondrocytes cultivated under normoxia or hypoxia. TNFα could not be induced by 2ME2 or DMSO either (data not shown).

#### Dimethyloxaloylglycine induces HIF-1-dependent genes but interferes with collagen synthesis

**Figure 6:**
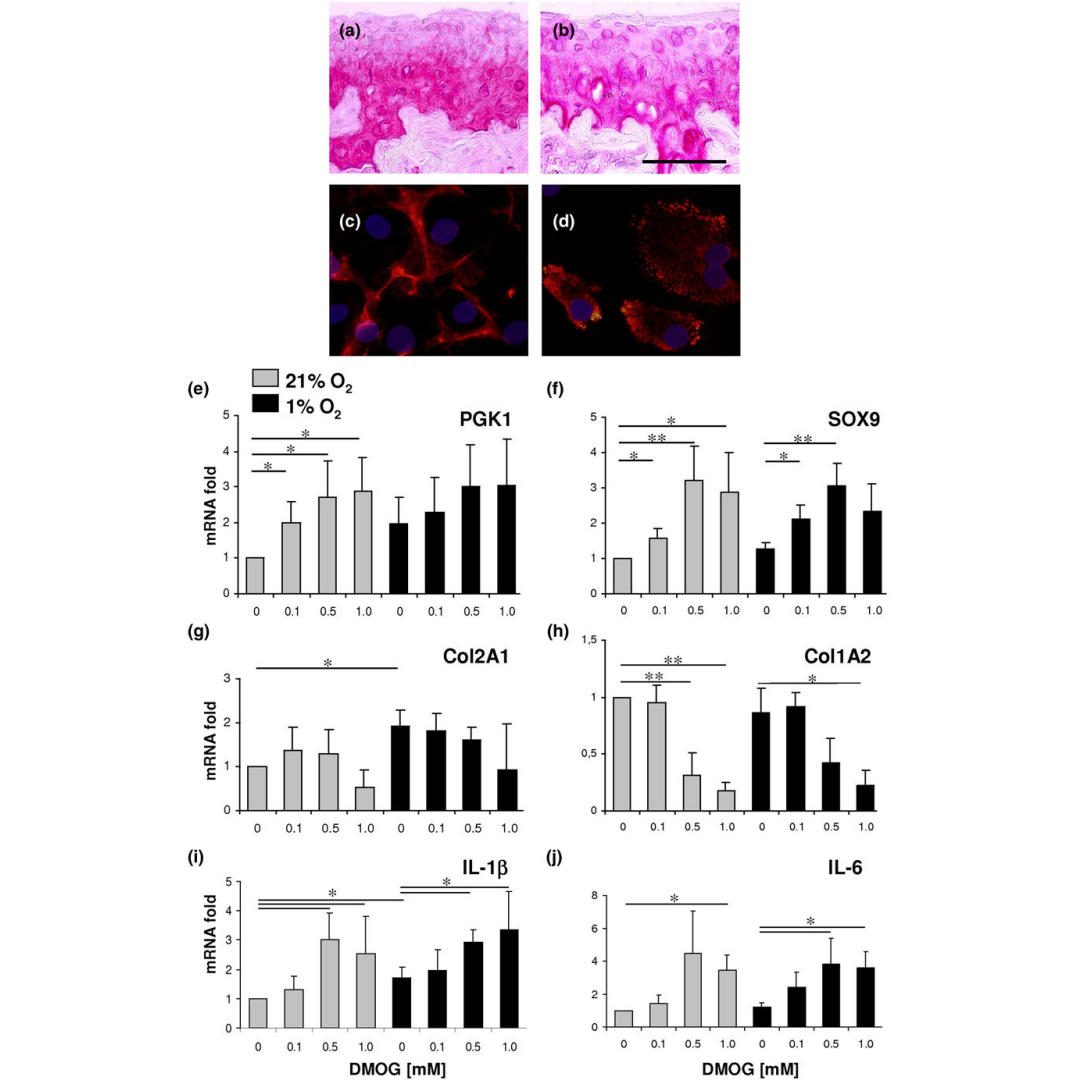
Biological effects of dimethyloxaloylglycine. In control joints of STR/ORT mice, **(a) **immunohistochemical detection of type II collagen reveals homogeneous staining within the extracellular matrix apart from some irregularities due to degenerative changes in the superficial layers. **(b) **In contrast, administration of dimethyloxaloylglycine (DMOG) results in accumulation of type II collagen located intracellularly/pericellularly in the superficial layer. **(c) **Type II collagen-immunofluorescence of cultured nontreated primary chondrocytes typically displays a pericellular network of type II collagen fibers. **(d) **DMOG treatment of primary chondrocytes in monolayer culture leads to an intracellular retention of vesicle-like structures that stain positive for type II collagen. Expression of **(e) **phosphoglycerate kinase 1 (PGK1), **(f) **Sox9, **(g) **type II collagen α_1_-chain (Col2A1), **(h) **type I collagen α_2_-chain (Col1A2), **(i) **IL-1β and **(j) **IL-6 in cultured articular chondrocytes determined by real-time RT-PCR dependent on treatment with or without DMOG under 21% or 1% oxygen. Expression levels are shown as the mean ± standard deviation. *P <0.05, **P <0.01. Bar = 100 μm.

Since DMOG had no visible positive effect on the progression of OA, further studies explored its biological effects – particularly its influence on the synthesis of type II collagen and the expression of cartilage-specific genes. Immunohistochemical staining for type II collagen differed from DMOG-treated joints to control joints. In the latter, type II collagen was distributed homogeneously throughout the extracellular matrix apart from some irregularities due to degenerative changes (Figure 6a). DMOG-treated joints, however, were characterized by an intracellularly and pericellularly pronounced staining pattern for type II collagen, particularly in the superficial half of the articular cartilage layer (Figure 6b). Similar observations were made in primary chondrocyte monolayer cultures employing immunofluorescence staining for type II collagen. As expected, nontreated chondrocytes typically secreted type II collagen to form an extracellular network (Figure 6c). On the contrary, addition of DMOG to the cultures leads to strong intracellular signals in the form of vesicle-like structures with virtually no or drastically reduced deposition of type II collagen in the extracellular space (Figure 6d).

We further investigated the effect of DMOG on the expression of two HIF-regulated genes, PGK1 and SOX9, using quantitative RT-PCR. Furthermore, the influences on the expression of Col1A2 and Col2A1 genes, which encode the structural elements for type I and type II collagen fibrils, were determined. DMOG significantly increased the expression of PGK1 in a dose-dependent manner (Figure 6e). This effect was more pronounced under normoxic conditions that are characterized by a physiologically lower activity of HIF-1. This supports the fact that the stabilization of HIF-1α by DMOG also increases its transactivating activity and thus secondarily increases the expression of HIF target genes. The expression of Sox9, a recently identified HIF target gene, was also stimulated in a dose-dependent manner by DMOG, both under normoxic and hypoxic culture conditions (Figure 6f).

Different results were observed, however, for the expression of Col1A2 and Col2A1. The transcription of Col2A1, which is a target of the transcription factor SOX9 and which encodes for cartilage-specific collagen fibers, was significantly increased under hypoxia but was not stimulated by DMOG. The highest concentration of DMOG (1 mM) even led to a measurable, although not significant, decrease in Col2A1 expression, both under normoxia and hypoxia (Figure 6g). Even more impressively, DMOG inhibited the expression of Col1A2 in a highly significant and dose-dependent manner irrespective of the oxygen status (Figure 6h).

DMOG induced the expression of the inflammatory cytokines IL-1β (Figure 6i) and IL-6 (Figure 6j) in a dose-dependent manner. DMOG did not, however, lead to a measurable enhanced expression of TNFα in articular chondrocytes (data not shown).

## Discussion

The present work documents the importance of the transcription factor HIF-1 for maintaining the integrity of hypoxic articular cartilage. The functional inhibition of HIF-1 coincided with increased apoptosis of articular chondrocytes and led to degenerative changes in murine knee joints. The study therefore supports the results of Schipani and colleagues obtained from conditional HIF-knockout mice in which cell death in the center of cartilaginous elements of the developing skeleton was observed [1]. Further studies confirmed that its subunit, HIF-1α, plays an important role in chondrogenesis and joint formation – and HIF-1α was recently shown to activate the promotor of SOX9, which is a key regulator of chondrocyte differentiation [3,4,23,24].

The naturally occurring derivate of estradiol, 2ME2, is considered a substance with anti-tumorigenic properties and is also accepted as a HIF inhibitor. Although its exact mechanism of action is not yet known, the HIF-1-inhibiting properties of 2ME2 have been suggested to be linked to a microtubule depolymerizing effect [14,25,26]. 2ME2 is a substance currently investigated in clinical trials for the treatment of tumors, and its systemic application was shown to be well tolerated with no relevant negative side effects in oxygenated organs [27]. *In vitro *studies also confirm that 2ME2 in moderate concentrations has no direct toxic effects on slow-dividing nontumor cells but does lead to a reliable inhibition of HIF-1 [12,13].

The present study has shown for the first time that repeated intraarticular administration of 2ME2 decreased the number and the intensity of HIF-1α-positive cells. The relevance of tissue hypoxia even in thin murine articular cartilage was demonstrated by the Hydroxyprobe™ (Chemicon) method, which detected hypoxic cells in deep and superficial layers of avascular cartilage and mensical tissue. This observation indicates the relevance of the transcription factor HIF-1 even in relatively thin murine articular cartilage. One can therefore conclude that 2ME2-exposed cells were no longer able to adapt to a hypoxic environment, resulting in impaired cell functions and cell death – which is presumably the primary pathomechanism for the degeneration of the cartilage tissue in this model. Of course, one has to consider that the molecular mechanism of cartilage degeneration induced by 2ME2 differs to some extent from the pathogenesis of primary OA, in which apoptotic cell death is supposed to play an inferior role [28]. Nevertheless, the morphological changes strikingly resembled those of osteoarthritic lesions. The lack of synovial inflammation and the absent induction of inflammatory cytokines suggest that cartilage destruction by 2ME2 does not depend on inflammatory reactions, but rather on an impact on the chondrocyte function itself.

The second objective of the present work was to investigate whether stabilization of HIF-1 could prevent the initiation or progression of murine OA in STR/ORT mice, which belong to a family of mouse strains that develop spontaneously degenerative changes [16,17,19]. In preceding *in vitro *experiments, DMOG was shown to effectively stabilize HIF-1 by inhibiting its degradation [12,15].

Despite the stimulatory effects on SOX9 expression *in vitro*, the repeated intra-articular injection of DMOG did not prevent the progression of OA in the knee joints of STR/ORT mice. Neither osteophyte formation nor the score for destruction of the articular cartilage differed between DMOG-treated joints and control joints. Degeneration of the articular cartilage and osteophyte formation were present in all knee compartments. Consistent with previous reports, cartilage degeneration was most prominent in the medial compartment. This phenomenon might be a consequence of mechanical overloading due to the varus deformity of the hind limbs [17,19].

The following three reasons may account for the failure of DMOG to have a beneficial effect. Firstly, endogenous HIF-1 levels may be sufficient in articular chondrocytes, and were even shown to be elevated in OA cartilage [9,11,29]. The data of the present study show that articular chondrocytes strictly depend on sufficient HIF-1 activity to ensure their cellular survival within the hypoxic matrix. Other studies on other cell types, however, describe a contradictory role of HIF-1α in modulating cell viability and apoptosis, which may depend on multitude factors including the cell type or the physico-chemical environment [30-32]. The physiological function of HIF-1α may therefore depend on a well-balanced activity, and surplus levels may not necessarily have further protective effects on the cells or even induce proapoptotic or other detrimental events that could not be detected by the TUNEL method used in the present study.

Secondly, DMOG represents rather a nonselective inhibitor of prolyl-hydroxylases. To our knowledge, agents that selectively inhibit the HIF-degrading prolyl-hydroxylase have not so far been established. DMOG not only inhibits the HIF prolyl-hydroxylase, but also interferes with collagen prolyl-hydroxylases [33]. The latter are involved in post-transcriptional processing of collagen fibers and formation of the triple helices. The hydroxylation of proline residues is essential for the formation of intramolecular hydrogen bonds and contributes to the stability of the triple-helical conformation [34]. The intracellular accumulation of type II collagen observed by immunohistochemistry and immunofluorescence can therefore be ascribed to impaired collagen processing, which is known to interfere with proper secretion of fibrillar collagens [35]. As a consequence, retention and accumulation of collagen molecules may act via a negative feedback loop on their gene expression. Downregulation of the collagen gene expression was more prominent for Col1A2 than for Col2A1. Two mechanisms may counteract specifically the expression of Col2A1. The described putative suppressive effect by intracellular collagen retention may at least partly be neutralized by a stimulatory effect via increased SOX9 expression, which is known to function as an enhancer for Col2A1 [36].

Thirdly, HIF-1α not only mediates prochondrogenic effects (for example, by enhanced SOX9 expression), but is also involved in catabolic events. HIF-1α was recently shown to be essential for myeloid cell-mediated inflammation [37]. Elevation of HIF-1α levels by DMOG increased the expression of the catabolic cytokines IL-1β and IL-6. IL-1β has recently been shown to be a HIF-1-target gene since its promotor region carries multiple HIF-1-binding sites resulting in an enhanced expression under hypoxia [38]. Although moderate doses of IL-1β also support anabolic effects on cartilage metabolism to some degree [39,40], it is well proven that this cytokine generally mediates catabolic events by canonical pathways and, therefore, may also account for the negative effect on collagen expression in the present study.

## Conclusion

The data from the present work underline the requirement of adequate levels of HIF-1 for the viability of chondrocytes and for the maintenance of the integrity of hypoxic articular cartilage. One has to consider, however, that surplus levels of HIF-1α may also exert catabolic effects – for example, by inducing the cytokines IL-1β and IL-6. HIF-1α may thus exert a paradoxical role in cartilage. On the one hand, HIF-1α ensures energy supply and survival of chondrocytes exposed to a hypoxic environment. Furthermore, HIF-1α stimulates the expression of a number of cartilage-specific genes. On the other hand, HIF-1α also promotes catabolic pathways and may also trigger proapoptotic events. In view of the data of the present study and the data from the literature, HIF-1α may be considered an important element in balancing anabolism and catabolism as well as cellular protection and cell death in cartilage and many other tissues. In this context, the question still remains open if elevated HIF-1α levels in OA exert a compensatory protective role or trigger the disease process.

In view of the detrimental side effect of DMOG on collagen metabolism due to its lack of specificity, one cannot currently rule out that more specific agents might nevertheless have beneficial effects. The future development of strictly selective inhibitors of HIF-degrading prolyl-hydroxylases therefore seems worthwhile, since stabilization of HIF may be of potential therapeutic value at least for cartilage repair approaches by supporting chondrogenic differentiation (for example, via stimulating expression of SOX9).

## Abbreviations

BSA: bovine serum albumin; Col1A2: type I collagen α_2_-chain; Col2A1: type II collagen α_1_-chain; DMEM: Dulbecco's modified Eagle's medium; DMOG: dimethyloxaloylglycine; DMSO: dimethyl sulfoxide; FCS: fetal calf serum; FITC: Fluorescein isothiocyanate; H&E: hematoxylin and eosin; HIF: hypoxia-inducible factor; IL: interleukin; 2ME2: 2-methoxyestradiol; OA: osteoarthritis; PBS: phosphate-buffered saline; PCR: polymerase chain reaction; PGK1: phosphoglycerate kinase 1; RT: reverse transcription; TNF: tumor necrosis factor; TUNEL: terminal deoxynucleotidyl transferase-mediated dUTP-biotin nick end labeling.

## Competing interests

The authors declare that they have no competing interests.

## Authors' contributions

KG carried out the study design, the acquisition of data, the analysis and interpretation of data and drafted the manuscript. DP participated in the study design and drafted the manuscript. SO and KXK were involved in the acquisition of the data. MW participated in the analysis and interpretation of data. FFH and BS conceived the study, participated in its design and coordination, and helped to draft the manuscript. All authors read and approved the final manuscript.
